# Critical appraisal of the piRNA-PIWI axis in cancer and cancer stem cells

**DOI:** 10.1186/s40364-024-00563-3

**Published:** 2024-02-01

**Authors:** Elena Garcia-Borja, Frantisek Siegl, Rosana Mateu, Ondrej Slaby, Aleksi Sedo, Petr Busek, Jiri Sana

**Affiliations:** 1 Laboratory of Cancer Cell Biology, Institute of Biochemistry and Experimental Oncology, First Faculty of Medicine, Charles University, U Nemocnice 478/5, Prague 2, 128 53 Czech Republic; 2 Central European Institute of Technology, Masaryk University, Kamenice 753/5, Brno, 625 00 Czech Republic; 3 Department of Biochemistry, Faculty of Science, Masaryk University, Brno, Czech Republic; 4 Department of Biology, Faculty of Medicine, Masaryk University, Brno, Czech Republic; 5 Department of Comprehensive Cancer Care, Masaryk Memorial Cancer Institute, Brno, Czech Republic; 6 Department of Pathology, University Hospital Brno, Brno, Czech Republic

**Keywords:** PIWI-interacting RNAs, piRNAs, PIWI proteins, Cancer stem cells, Cancer

## Abstract

Small noncoding RNAs play an important role in various disease states, including cancer. PIWI proteins, a subfamily of Argonaute proteins, and PIWI-interacting RNAs (piRNAs) were originally described as germline-specific molecules that inhibit the deleterious activity of transposable elements. However, several studies have suggested a role for the piRNA-PIWI axis in somatic cells, including somatic stem cells. Dysregulated expression of piRNAs and PIWI proteins in human tumors implies that, analogously to their roles in undifferentiated cells under physiological conditions, these molecules may be important for cancer stem cells and thus contribute to cancer progression. We provide an overview of piRNA biogenesis and critically review the evidence for the role of piRNA-PIWI axis in cancer stem cells. In addition, we examine the potential of piRNAs and PIWI proteins to become biomarkers in cancer.

## Background

Non-coding RNAs play an important role in epigenetic and post-transcriptional gene regulation, having a profound effect on the complex orchestration of gene expression. A new class of small non-coding RNAs, known as PIWI-interacting RNAs (piRNAs), has emerged as an intriguing subject of investigation in the realm of gene regulation. PiRNAs were initially recognized for their role in germline cells. Through the formation of piRNA-induced silencing complex (piRISC) with PIWI proteins, piRNAs control the potentially harmful activities of transposable elements within the genome [1]. The threat posed by transposition lies in its capacity to disrupt genomic stability, potentially leading to mutations, double-strand breaks and even serving as an initial step in cancer development. Recently, piRNAs have also piqued researchers’ interest due to their presence and potential roles in somatic cells, particularly in cancer cells, where their actions may diverge from those in germ cells.

An expanding body of research has revealed dysregulation of piRNAs and PIWI proteins in various types of tumors. However, a compelling question remains: is this dysregulation the primary cause of cancer development or a consequence thereof? As our understanding deepens, it becomes increasingly evident that altered expression of piRNAs and PIWI proteins can significantly impact the behavior and characteristics of somatic cells as well as cancer cells, including cancer stem cells. This suggests that the role of the piRNA-PIWI axis extends beyond germ cells. The expanding body of evidence underscores the significance of the piRNA-PIWI axis in the complex field of tumor biology, positioning it as a promising area of investigation that could provide new insights into cancer pathogenesis and therapy.

However, despite a growing number of studies, consistent conclusions have not yet been reached. Therefore, this review aims to critically summarize the current knowledge in this area and thus help further research on piRNA and PIWI molecules in cancer, including cancer stem cells.

## piRNAs and PIWI proteins

PiRNAs are small noncoding RNAs (sncRNAs) that were discovered in 2001 in *Drosophila melanogaster* [2]. PiRNAs are highly conserved in all bilaterian animals and play essential biological roles, especially in the germline [3, 4]. Across different species, the length of piRNAs varies from 21 up to 35 nucleotides [3, 5]. A defining characteristic of piRNAs is their interaction with a germline-specific subfamily of Argonaute RNA-binding proteins known as P-element Induced Wimpy testis (PIWI) proteins (Table 1). The PIWI proteins are highly conserved across species. The four PIWI proteins in humans are PIWIL1, PIWIL2, PIWIL3, and PIWIL4. In *Mus musculus*, the corresponding homologous PIWI proteins are MIWI, MILI, and MIWI2, while in *Drosophila melanogaster*, they are Piwi, Aub, and Ago3 [6]. PIWI proteins are highly basic and have a molecular weight ranging between 96 and 110 kDa. Like other Argonaute proteins, PIWI proteins contain a conserved PAZ (PIWI/Argonaute/Zwille) and MID domain, which recognize the 3’ and 5’ end of piRNA intermediates, respectively [7]. A unique domain called PIWI functions as an RNase H domain and is responsible for piRNA-guided hydrolysis of single-stranded RNAs. Alternatively spliced transcript variants encoding different protein isoforms have been described. However, the functional implications of the distinct protein isoforms remain unknown.

**Table 1:** Characteristics of human PIWI proteins

	**PIWIL1**	**PIWIL2**	**PIWIL3**	**PIWIL4**
**Other names**	HIWI	HILI	HIWI3	HIWI2
**Gene location**	12q24.33	8p21.3	22q11.23	11q21
**Number of exons**	26	25	23	20
**Protein isoforms (transcript variants)**	Isoform 1 (tv1) Isoform 2 (tv2)	Isoform 1 (tv1 and tv2) Isoform 2 (tv3)	Isoform 1 (tv1) Isoform 2 (tv2)	Isoform 1 (tv1)
**Number of amino acids**	861 (isoform 1)	973 (isoform 1)	882 (isoform 1)	852 (isoform 1)
**Molecular weight (kDa)**	98.6 (isoform 1)	109.8 (isoform 1)	101.1 (isoform 1)	96.6 (isoform 1)
**Isoelectric point**	9.258 (isoform 1)	8.781 (isoform 1)	9.328 (isoform 1)	8.669 (isoform 1)

The information was extracted from GenBank and UniProt databases. The isoelectric point was calculated with the Prot pi calculator. *Tv* transcript variant

PiRNAs together with PIWI proteins form a piRISC capable of targeting nascent transcripts of transposable elements (TE) [8] protecting the genome of germ stem cells [3, 4]. Although in most arthropods and mollusks, piRNAs and PIWI proteins can be expressed in somatic tissues [9, 10], in vertebrates PIWI proteins are only highly expressed in gonads, while their expression is usually absent in other somatic tissues [11–16]. In addition to this most prominent role, piRISCs are involved in the regulation of gene expression by targeting specific mRNAs in a microRNA-like manner, epigenetic regulation of TE loci, and in defense against viral infections [3, 8, 17].

## Biogenesis of piRNAs

Models of piRNA biogenesis are inconsistent in different studies, which could be partially explained by the differences among species. Another layer of complexity of piRNA biogenesis lies at the cellular level, as different cell types can activate the transcription of specific piRNA clusters and generate piRNAs by distinct mechanisms [18]. The main differences from other classes of sncRNA are that piRNA biogenesis is Dicer-independent, occurs only on single-stranded precursors, and mature molecules are loaded onto PIWI proteins [19].

### Transcription of piRNA loci

**Fig. 1:**
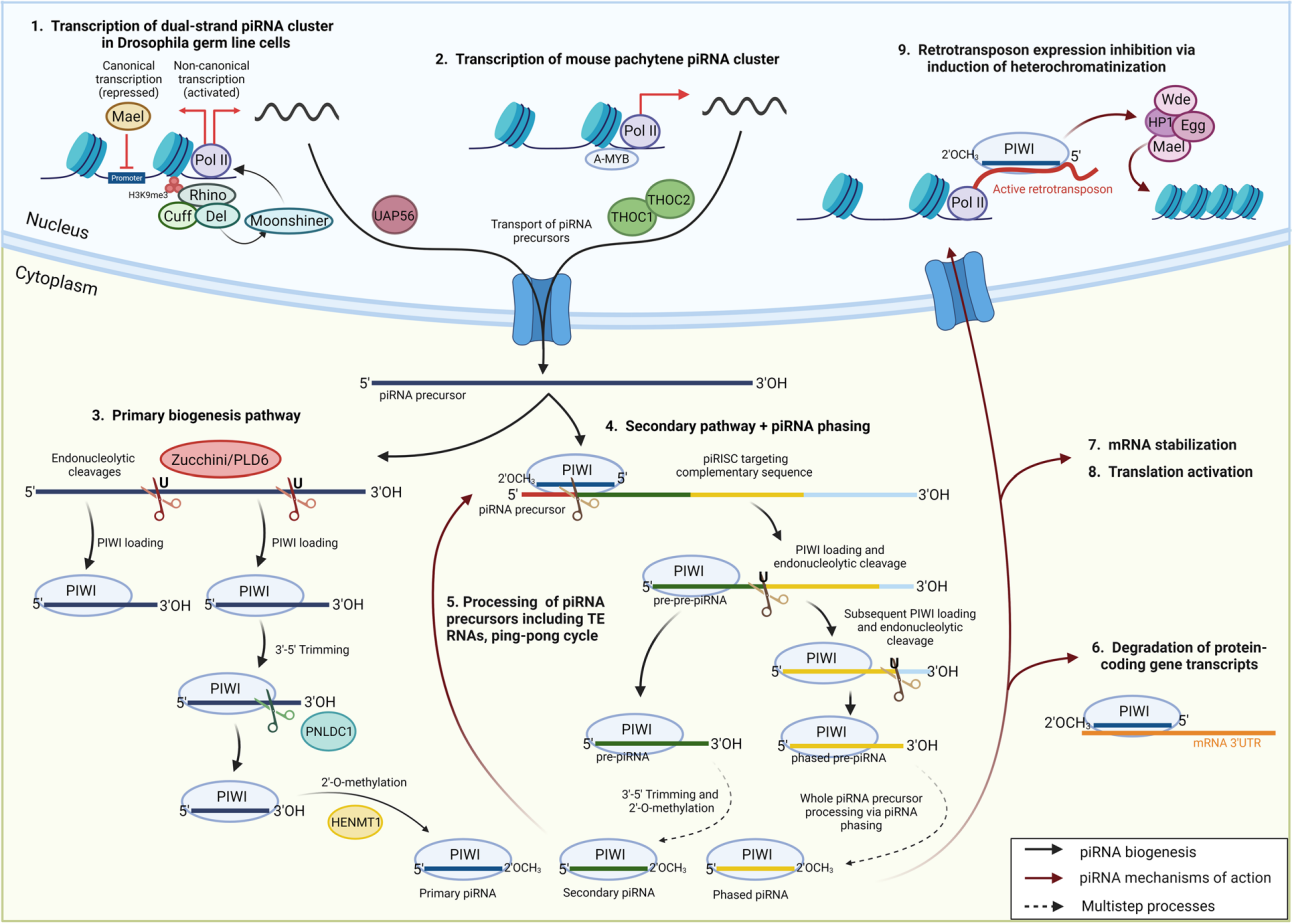
Schematic representation of piRNA biogenesis and mechanisms of action. (1) In Drosophila, dual-strand piRNA clusters are marked by H3K9me3, which are targeted by Rhino. Subsequently, Rhino together with Deadlock and Cutoff recruits Moonshiner, leading to the activation of non-canonical transcription via RNA polymerase II. Canonical transcription at the dual-strand piRNA clusters is repressed by Maelstrom (Mael). UAP56 ensures the transport of piRNA precursors into the cytoplasm. (2) In mice, A-MYB recognizes piRNA clusters and ensures transcription catalyzed by RNA polymerase II. THOC1, and THOC2 proteins are responsible for the cytoplasmic transport of piRNA precursors. (3) In the cytoplasm, piRNA precursors undergo primary biogenetic pathway consisting of endonucleolytic cleavage by Zucchini/PLD6, PIWI loading, 3’ end trimming by PNLDC1 and 2’-O-methylation by HENMT1. (4) According to a unified model, the piRNA precursor is targeted by a piRISC with a complementary piRNA leading to the cleavage and generation of a new 5’ end. Subsequently, another PIWI protein is loaded and guides an endonuclease that cleaves the precursor. The resulting pre-piRNA is 3’ trimmed and 2’-O-methylated. The remaining piRNA precursor is repeatedly processed in the same manner, leading to the production of a variety of phased piRNAs. (5) Mature piRISCs target complementary piRNA precursors and transposon RNA transcripts, leading to their cleavage and further processing including amplification through the “ping-pong “ cycle. (6) piRISCs can also degrade protein-coding gene transcripts by targeting complementary sequences in the 3’ UTR of mRNAs in a miRNA-like manner. Alternatively, this interaction can (7) stabilize mRNAs or (8) activate translation. (9) In the nucleus, piRISCs target active transposons and repress their transcription by recruiting epigenetic machinery, which induces heterochromatinization. Created with BioRender.com

The biogenesis of piRNAs starts in the nucleus with the transcription of piRNA clusters into single-stranded piRNA precursors [20] (Fig. 1). Based on the location of the piRNA cluster, the offspring molecules can be classified as TE-derived piRNAs, 3’UTR piRNAs, or intergenic piRNAs [21]. Most piRNA clusters are marked by a repressive H3K9me3 and reside in the heterochromatin. This mark is required to ensure the recruitment of Rhino (an HP1 paralogue), Cutoff (Cuff), and Deadlock proteins in Drosophila dual-strand clusters [22, 23]. Rhino together with Deadlock recruits Moonshiner and RNA polymerase II, leading to the formation of a transcription pre-initiation complex [24]. Thus, a paradoxical situation occurs as the loci that should be silenced by H3K9me3 are predestined for transcription. The specific function of Cutoff is rather uncertain, although it seems that this protein is important for the protection of uncapped 5’ends of bidirectional cluster transcripts [23, 25, 26]. In addition, Maelstrom (Mael) plays an important role in the transcription of piRNA clusters. When Rhino recruits transcription factors, including RNA Pol II for non-canonical transcription in dual-strand piRNA clusters, Mael prevents canonical promoter dependent transcription at these sites, which would result in low piRNA processing rate [27]. In mammals, predestination of piRNA clusters for transcription is more complicated, as no molecular complex responsible for the recruitment of the transcription machinery has been described [22]. Certain mammalian piRNA precursors originate from genomic regions that are not under the control of promoters and are highly repetitive and abundant in TE sequences. On the other hand, other piRNA precursors originate from long noncoding RNAs with a 5’ cap and a 3’ polyA tail, not associated with TEs [24, 28]. The only mechanism of transcription regulation in mammals has been described for mouse pachytene piRNA clusters. These clusters are controlled by the A-MYB protein, which is essential for the transcription of the entire population of pachytene piRNAs. In addition, A-MYB is important for the transcription of specific components of the piRNA biogenesis pathway such as PIWIL1 (MIWI) or TDRD1 [28].

PiRNA precursors are transported from the nucleus to the cytoplasm, where the rest of the piRNA biogenesis occurs. In Drosophila, the transport involves UAP56, a nuclear DEAD box protein that interacts with Rhino and the piRNA precursors, and Vasa, which is indispensable for the transport of piRNA precursors to the nuage [29]. In mammals, subunits of the THO complex, THOC1 and THOC2, bind pachytene piRNA precursors and contribute to their export into the cytosol [30]. However, the exact transport mechanism for most piRNA precursors in mammals is unknown.

### Processing of piRNA precursors

PiRNA precursors entering the cytoplasm are processed via the piRNA biogenetic machinery to produce mature piRISCs (see [1] for comprehensive review). Originally, it was believed that two biogenetic pathways – primary and secondary (also called “ping-pong” cycle) – are employed to generate and maintain a stable pool of piRNAs [31]. After leaving the nucleus, piRNA precursors are transported to the mitochondrial membrane, where they are cleaved by Zucchini in Drosophila; the human homologue remains unknown [32]. The cleaved fragments of piRNA precursors are subsequently loaded onto PIWI proteins, and the 3’ end is processed by the exonuclease PNDLC1 and 2’-O methylated by HENMT1 [33]. It was thought that the primary piRNAs generated by this pathway serve as guides for further processing of piRNA precursors and generation of secondary piRNAs, thus maintaining a stable pool of piRNAs via the “ping-pong “ cycle [34].

A unified model has recently been proposed, where the primary pathway generates piRISCs, which initiate the processing of the piRNA precursors. PiRISC catalyzes the cleavage and formation of a new 5’ end of the piRNA precursor, now termed pre-pre-piRNA. Following this, a PIWI protein is loaded onto the 5’ end of the pre-pre-piRNA. The PIWI protein then guides an endonuclease that cleaves the pre-pre-piRNA at the first uridine downstream of the bound sequence resulting in the formation of a PIWI protein–pre-piRNA complex and the release of a shortened pre-pre-piRNA with a new 5’ end. This pre-pre-piRNA is subsequently bound by another PIWI protein. Repeating this process leads to the successive processing of the whole piRNA precursor [35], which is referred to as piRNA phasing or trailing and leads to a massive increase in sequence variability of piRNAs. The 3’-5’ exoribonuclease PNDLC1 adjusts the pre-piRNA bound to a particular PIWI protein [36]. Finally, the S-adenosylmethionin-dependent methyltransferase HENMT1 methylates the 2’hydroxyl group at the 3’ end [33], protecting the piRNAs from degradation [37]. This processing finalizes the maturation of piRNAs leading to mature piRISCs.

## Mechanisms of action of piRNAs and PIWI proteins

PiRISCs play essential roles in animal germline development, supporting germ cell maintenance and gamete differentiation. More recent studies have focused on their function in somatic cells, in particular in cancer cells. Most piRNAs are complementary to TE sequences, suggesting that TEs are their primary target [38]. However, piRISCs not only control TE mobilization, but also affect the expression of protein coding genes and lncRNAs [39].

A piRISC is guided by the interaction of its piRNA with a complementary nucleic acid sequence, triggering the recruitment of machinery that will lead to histone modification, methylation of DNA, or post-transcriptional regulation. Additionally, PIWI proteins may interact with other proteins independent of piRNA-nucleic acid pairing. These protein–protein interactions may lead among others to phosphorylation and ubiquitination [7, 39–41].

### Chromatin remodeling

#### Histone modification

PiRISCs can enter the nucleus and act as epigenetic silencers through the recruitment of chromatin-modifying enzymes [41]. In Drosophila, piRISCs interact with the zinc finger protein Asterix initiating TE surveillance [42]. In this process, the piRNAs recognize their complementary targets and the piRISCs interact with Panoramix (also called Silencio) that recruits the transcriptional repression machinery. Firstly, Eggless (Egg) and its cofactor Windei (Wde) catalyze the formation of H3K9me3 and subsequent recruitment of HP1 leads to heterochromatin formation [43]. In addition, Lysine-specific demethylase 1 (Lsd1) likely removes activating H3K4me2 marks from transposon promoter regions leading to transcription silencing [44]. In mice, a similar mechanism is observed, as Miwi2-piRISC targets nascent transcripts to silence active transposons via the trimethylation of H3K9, leading to heterechromatinization and transcription inactivation. Interestingly, this mechanism was observed only for full-length, actively transcribed copies of LINE transposons. 5’-truncated LINEs, which are not transcribed, are not targeted by the piRNA machinery [45].

Maelstrom (Mael), a putative single-strand RNA-binding protein, is the final effector of piRISC-induced heterochromatinization. It is required for transcriptional silencing, but its depletion does not lead to the reduction of H3K9me3 marks, but rather to their spread into downstream regions [44].

A similar mechanism may silence the expression of tumor suppressors in cancer cells. In leukemia cells, piRNA hsa_piR_011186 recruits histone methyltransferases downregulating the expression of CDKN2B upon binding to a complementary sequence. This results in increased cell proliferation and inhibition of apoptosis [46]. On the other hand, a piRNA derived from a tumor-suppressive lncRNA and PIWIL1/4 recruit histone-modifying enzymes MLL3 and UTX (or KDM6A) to activate the transcription of TRAIL (TNF-related apoptosis-inducing ligand) leading to tumor growth inhibition [47].

#### DNA methylation

PiRISCs can induce methylation of DNA by recruiting DNA methyltransferases (DNMT) [48]. In mice, PIWIL2 (MILI) and PIWIL4 (MIWI2) play an essential role in de novo DNA methylation of TEs [6, 41, 48, 49].

Despite the high evolutionary conservation of this function [50], the exact mechanism is poorly understood. SPOCD1, a PIWIL4 (MIWI2)-associated factor, is exclusively expressed during the period of de novo methylation of the genome. It interacts with epigenetic machinery proteins such as DNMT3L, DNMT3A, NURD or BAF leading to the silencing of TEs. The interaction of SPOCD1 and target nascent TEs seems to be guided by a piRISC [51]. Recently, the PIWIL4 (MIWI2) piRISC together with MORC3 has been shown to promote de novo DNA methylation leading to TE silencing in embryonic testes [52].

It was further suggested that the piRNA-PIWI axis had a role in silencing of non-TE loci in cancer cells [53, 54]. For example, piRNA-823 contributes to aberrant DNA methylation of cell cycle regulators and apoptosis-related proteins in multiple myeloma [55]. Moreover, PIWIL1 expression is associated with DNA methylation in sarcoma, and its downregulation reduces global DNA methylation and suppresses tumorigenesis [6].

### Post-transcriptional regulation

PiRNAs can directly target the 3'UTR of mRNAs and regulate their expression through posttranscriptional regulation [7, 56]. Similarly to miRISCs, piRISCs usually target the mRNAs through perfect pairing of nucleotides in the seed region at the 5'end and have less stringent requirements for complementarity near the 3'end [56, 57]. Nevertheless, for highly abundant piRNAs in complex with PIWIL1 or PIWL2, perfect complementarity in the seed region is not required [58]. On the one hand, piRISCs binding can result in mRNA cleavage and decay. On the other hand, piRISCs can stabilize target mRNAs by promoting their polyadenylation or activate translation by interacting with initiation factors [59].

#### mRNA cleavage and decay

Several studies described that the piRNA-PIWI axis could be involved in the degradation of protein-coding transcripts. In the soma of Drosophila embryo, the PIWI protein Aub can either directly cleave target mRNAs or facilitate their deadenylation and subsequent decay. Mechanistically, Aub interacts with the RNA-binding protein Smaug to recruit the deadenylating CCR4-NOT complex [60, 61].

In mouse late spermatocytes, piRNAs originated from TEs and pseudogenes posttranscriptionally regulate many lncRNAs and mRNAs. Retrotransposons are present in the 3’ regions of many genes, enabling the piRNA-mediated regulation of their expression. Pseudogene-derived piRNAs potentially exert similar functions as they may be able to target the mRNA of their functional cognate gene. At least in part, the slicer activity of PIWIL1 contributes to the piRNA-mediated degradation of mRNAs and lncRNAs in the mouse testis [39]. Later in mouse spermiogenesis (in elongating spermatids), PIWIL1 guided by the pachytene piRNAs is responsible for the decay of protein-coding mRNAs through the recruitment of the deadenylase CAF1 [62]. Influence of pachytene piRNA expression on male fertility in mice is further demonstrated by the fertility defects following disruption of expression of specific pachytene piRNA clusters. Although thousands of piRNAs are derived from pi6 and pi18 pachytene clusters, only a few several dozen genes are dysregulated when their expression is disrupted. This also suggests specific piRNA-mRNA targeting mechanisms that differ from the miRNA-like mode, where a single miRNA can target vast number of mRNAs due to the short seed region. It is not yet clear whether this mechanism is somehow specific for pachytene piRNAs or whether it is a shared feature among piRNAs [63, 64].

In humans, these mechanisms are much less described. In lung cancer, piRNA-55490 can bind to the 3’-UTR of mammalian target of rapamycin (mTOR) mRNA leading to its degradation and resulting in the inhibition of cell proliferation. Accordingly, low levels of piRNA-55490 were associated with worse clinical outcome [65].

#### mRNA stabilization and translation activation

PIWIL1 and PIWIL2 were reported to interact with the cytoplasmic mRNA cap-binding complex [16, 66]. In mouse round spermatids, several transcripts are targeted through imperfect base-pairing with piRNAs bound to PIWIL1. PIWIL1 subsequently forms a complex with HuR, eIF3f, and poly(A)-binding protein (PABPC1), which activates translation of the target mRNA [67].

Similarly, in the germ plasm of Drosophila embryo, Aub targets mRNAs through imperfect base-pairing and activates translation through the recruitment of the poly(A)-binding protein (PABP) and the eIF3 initiation complex [68]. Alternatively, Aub interacts with the germline-specific cytoplasmic poly(A) polymerase Wispy to induce polyadenylation and stabilization of the target mRNA. The transition from mRNA decay in the soma to mRNA stabilization in the germ plasm might be due to the interaction of Aub with the proteins Tudor, Vasa, and Oskar, which are found in the germ granules. This transition may facilitate embryonic patterning in Drosophila since Aub mediates the degradation of *nanos* mRNA by deadenylation in the soma, while it stabilizes and activates its translation in the germ plasm [59, 69].

### piRNA binding-independent functions of PIWI proteins

Independent of piRNA base-pairing, PIWI proteins can participate in the posttranslational regulation of various target proteins. In mouse germ cells, MIWI (PIWIL1) regulates the histone-to-protamine exchange during spermiogenesis. PIWIL1 ubiquitination and subsequent degradation enables the E3 ubiquitin ligase RNF8 to enter the nucleus, facilitating histone ubiquitination. This process leads to histone-to-protamine exchange, which is essential for chromatin compaction and spermatid maturation [70].

The piRNA binding-independent functions of PIWI proteins may also explain some of the findings in human tumors, where PIWI overexpression does not always correspond with piRNA induction and formation of a piRISC [71, 72]. In pancreatic cancer cells, piRNAs were not detectable although PIWIL1 was aberrantly expressed. In the absence of piRNAs, PIWIL1 activated the anaphase promoting complex/cyclosome (APC/C), an E3 ubiquitin ligase that targets a cell adhesion-related protein Pinin, enhancing metastasis. In contrast to this pathological function, piRNA-loaded PIWIL1 is degraded after polyubiquitination by APC/C in late spermatids. Thus, depending on the presence of piRNAs, PIWIL1 serves as a co-activator, or a substrate of APC/C [72]. In colorectal cancer, PIWIL2 has been shown to interact with STAT3 and phospho-SRC leading to STAT3 phosphorylation and an increase in the proliferation, metastasis and chemoresistance of colorectal cancer cells [73]. In a piRNA-independent manner, PIWIL1 was also shown to interact with various components of the nonsense-mediated mRNA decay machinery such as UPF1 in gastric cancer. This interaction was essential for the regulation of mRNAs involved in cell cycle and cell adhesion, leading to increased cell proliferation and migration [74].

## Expression and possible role of piRNAs and PIWI proteins in cancer stem cells

Several studies have shown that piRNA and PIWI proteins are dysregulated in human cancers. It is currently believed that in both hematological and many types of solid tumors, there is a unique and relatively small subpopulation of undifferentiated and self-renewing cancer cells that drives tumor progression. These cells, known as cancer stem cells (CSC) or tumor initiating cells, possibly originate from non-malignant stem cells or progenitor cells, with which they share several characteristics, including the expression of stem cell markers such as SOX2, NANOG, CD133, and CD44. CSCs are capable of giving rise to more differentiated progenies, thus contributing to tumor heterogeneity. They are also thought to be critical for cancer metastasis and recurrence after treatment due to their intrinsic chemo- and radioresistance [75, 76]. Several processes, including epigenetic regulations, are important for the maintenance of their stem cell properties [77].

Since the piRNA-PIWI axis is more active in undifferentiated cells than in differentiated ones, regulates epigenetic processes, and is required for stem cell renewal, there is an intriguing possibility that it may be responsible for the maintenance, propagation, and survival of CSCs. The manipulation of this axis may thus represent a novel avenue for targeting a subpopulation of cancer cells crucial to tumor progression, resistance to treatment, and recurrence.

### piRNAs in cancer stem cells

Only a limited number of studies identified piRNAs associated with CSCs and/or an undifferentiated phenotype of cancer cells. In CD44^+^ /CD24^-^ breast CSCs, epithelial–mesenchymal transition (EMT) induction increased the levels of piR-932, which interacted with PIWIL2. However, the function of the PIWIL2/piR-932 piRISC has not been described [78].

Upon overexpression of PIWIL2 in fibroblasts, the cells acquired several characteristics of cancer stem-like cells and upregulated the expression of several piRNAs including piRNA MW557525. Inhibitors of piRNA MW557525 induced apoptosis and suppressed cell proliferation, migration and invasion and the expression of stem cell markers such as CD24, CD133, KLF4, and SOX2. NOP56, a protein involved in ribosomal biogenesis, was identified as a possible target gene of piRNA MW557525 mediating these effects [79].

Breast tumor samples showed higher expression of piR-823 as compared to paired healthy tissues. In addition, piR-823 was upregulated in the stem-like subpopulation of breast cancer cell lines as well as in CSCs isolated from breast cancer tissue. piR-823 overexpression caused increased proliferation and overexpression of stemness genes POU5F1, SOX2, KLF4, NANOG, and h-TERT. piR-823 also promoted the expression of DNA methyltransferases DNMT1, DNMT3A and DNMT3B, leading to the methylation of the *APC* gene and subsequent activation of Wnt signaling inducing the CSC phenotype. Accordingly, knockdown of piR-823 led to decreased proliferation, colony-forming capacity, and downregulation of the stemness genes. Notably, treatment with anti-piR-823 inhibited tumor growth [80].

piR-2158 was recently described as downregulated in breast cancer, with even more profound downregulation in ALDH^+^ breast CSCs compared to ALDH^-^ breast cancer cells. Overexpression of piR-2158 led to lower proliferation, migration and invasiveness, as well as decreased expression of EMT markers Vimentin, Fibronectin, ZEB1 and Slug. Remarkably, CSC markers, SOX2, OCT4, NANOG and KLF4, were also downregulated upon piR-2158 mimetics treatment. Results were replicated in vivo as the stable upregulation of piR-2158 led to suppressed tumor growth and angiogenesis. piR-2158 affects breast cancer biology via the targeting of IL11, leading to lower phosphorylation of STAT3 and impairment of JAK/STAT3 pathway, responsible for the dysregulation of angiogenesis and self-renewal of breast CSC [81].

### PIWIL1 in cancer stem cells

**Fig. 2:**
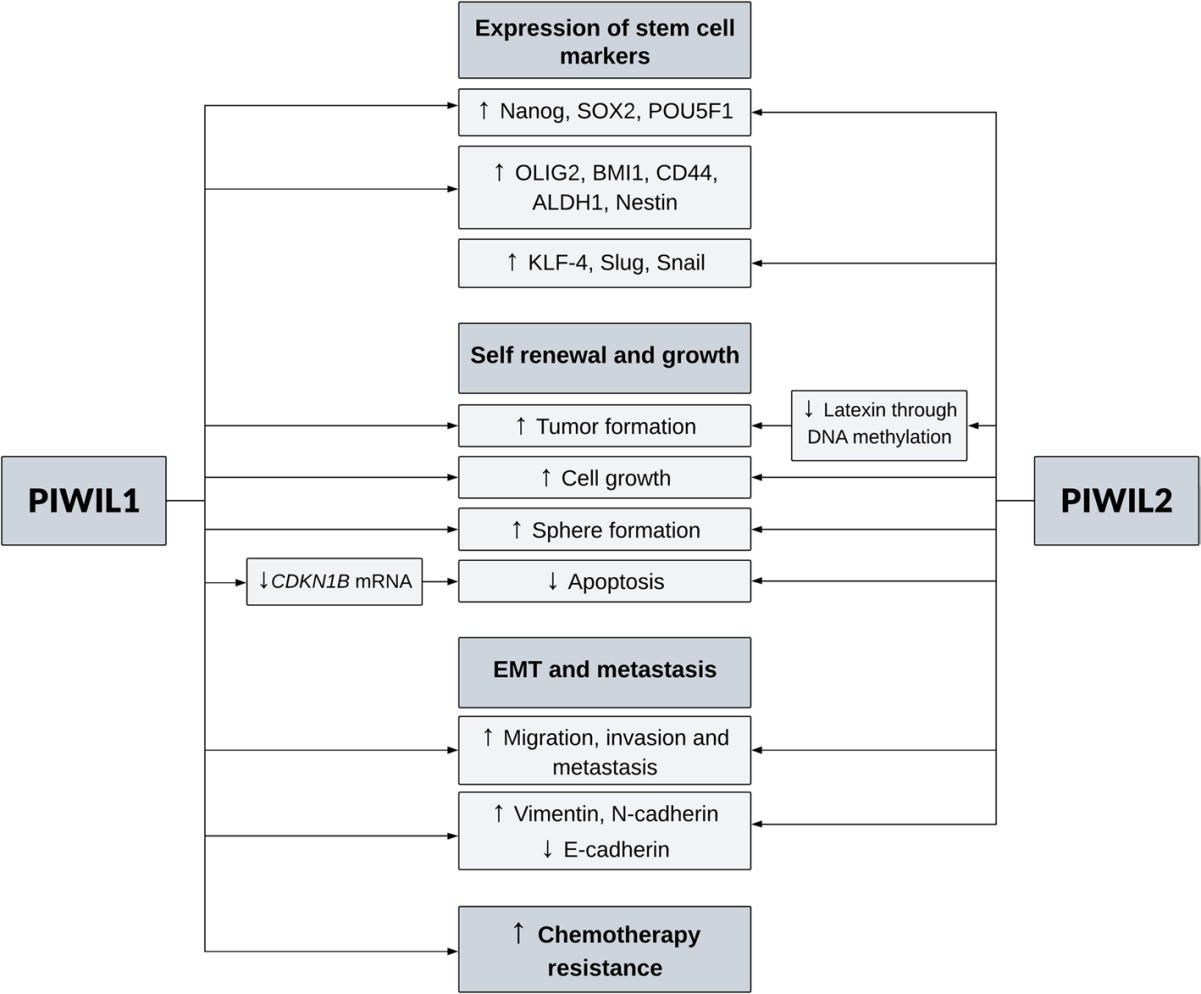
Effect of PIWI proteins on stem cell characteristics in cancer. PIWIL1 and PIWIL2 are linked to expression of stem cell markers, involved in cancer stem cell renewal and growth as well as epithelial mesenchymal transition (EMT) and resistance to chemotherapy. See text for details. CDKN1B = cyclin-dependent kinase inhibitor 1B, POU5F1 (Oct-3, Oct4, Oct3/4) = octamer-binding transcription factor 4, BMI-1 = polycomb complex protein BMI-1, ALDH1 = aldehyde dehydrogenase 1 family member A1, KLF-4 = Kruppel-like factor 4

Several studies have demonstrated a link between the expression of PIWIL1 and CSC markers (Fig. 2). On the tissue level, PIWIL1 expression was associated with a stem cell gene signature in non-small cell lung cancer [82], and expression of the stem cell markers POU5F1 (Oct3/4) and SOX2 and OLIG2 in colorectal cancer [83] and glioblastoma [84], respectively. Moreover, a study reported that PIWIL1 was expressed at higher levels in glioblastoma stem cells than in paired non-stem cells [84].

These correlative data are supported by studies examining the effects of PIWIL1 depletion or overexpression. In cervical cancer cells, PIWIL1 overexpression leads to increased expression of stem cell markers POU5F1, NANOG and BMI1, while PIWIL1 downregulation has the opposite effect [85]. Similar phenomena were observed for the stem cell markers CD44, ALDH1, POU5F1 and NANOG in endometrial cancer cells [86]. The upregulation of PIWIL1 led to an increase, while its downregulation resulted in a decrease in the side population and expression of the stemness genes such as *NANOG*, *SOX2*, and *POU5F1* in human myeloma cells [87]. Finally, PIWIL1 knockdown in glioblastoma decreased the expression of neural stem and progenitor cell markers OLIG2 and Nestin [84].

In functional assays, PIWIL1 promoted cell viability, tumor sphere formation in vitro and tumorigenesis in vivo in cervical and endometrial cancer and glioblastoma [84–86]. The underlying molecular mechanisms remain largely unknown, but a study in glioblastoma stem cells revealed that PIWIL1 downregulation reduced c-MYC levels and strongly increased BTG2 (B cell translocation gene 2), a tumor suppressor, and FBXW7 (F-Box WD Repeat Domain Containing 7), a component of the SCF (Skp, Cullin, F-box containing) complex, which regulates the stability of proteins such as c-MYC. Downregulation of PIWIL1 also increased the expression of CDKN1B, a cyclin dependent kinase that regulates G1 progression, and reduced the expression of cyclin D2 (CCND2), which regulates G1/S transition. As a result, PIWIL1 downregulation reduced the number of glioblastoma stem cells in the S phase. PIWIL1 knockdown also reduced the expression of myeloid leukemia cell differentiation protein (MCL1), induced apoptosis and facilitated senescence [84]. EMT (epithelial mesenchymal transition) promotes stem cell-like characteristics of cancer cells and is known to play a critical role in tumor metastasis and recurrence [88]. PIWIL1 overexpression in endometrial cancer cells promoted migration and invasion, possibly by inducing EMT [86].

There is a correlation between stemness, autophagy and drug resistance [89]. In myeloma cells, PIWIL1 overexpression resulted in higher cell viability and resistance to bortezomib, dexamethasone, and doxorubicin in vitro due to the activation of autophagy and mitophagy. In line with that, PIWIL1 downregulation decreased cell viability and sensitized the cells to cytotoxic drugs. The levels of mTOR and AKT-Ser473 were decreased in PIWIL1-overexpressing cells, while Parkin, optineurin and p-TBK-1, the proteins involved in mitophagy, were increased. Opposite results were observed in PIWIL1-downregulated cells [87].

### PIWIL2 in cancer stem cells

Similar to PIWIL1, several studies have indicated an association between PIWIL2 and cancer cell stemness (Fig. 2). Sphere-forming cells derived from cervical cancer expressed *PIWIL2* and stem cell-related genes such as *POU5F1*, *c-MYC*, *STAT3*, *SOX2* and exhibited stem cell properties, such as tumor formation, absence of CD34 and CD105 and expression of CD44, a marker of CSCs, and ABCG2, which conferred them resistance to doxorubicin [90]. Precancerous stem cells from mouse dendritic cell-like leukemia expressed embryonic and adult stem cell-related genes, were able to partially differentiate, had long-term repopulating activity, and could form tumors in adult mice. Downregulation of PIWI2 decreased the proliferation of these cells [91]. CD44^+^/CD24^−/low^ CSCs from breast cancer cell lines formed mammospheres and expressed PIWIL2 [12]. In a cohort of 782 breast cancer patients, *PIWIL2* correlated with genes involved in germ cell and stem cell proliferation and differentiation, and regulators of apoptosis belonging to the Bcl family [12]. Furthermore, a stable breast cancer cell line overexpressing PIWIL2 exhibited high POU5F1 and NANOG expression, indicating the embryonic stem-like identity of the PIWIL2-expressing population [12].

PIWIL2 overexpression in mouse embryonic fibroblasts induced an increase in growth, invasion capacity, an increased yield of lactate per glucose, and tolerance to hypoxia [92]. Foreskin fibroblasts transfected with PIWIL2-GFP changed their morphology from a typical long spindle shape to a small spherical shape and expressed stem cell markers such as POU5FI, NANOG, SOX2, KLF-4, and c-MYC, as well as endoderm markers [93]. PIWIL2 silencing in precancerous stem cells from mouse dendritic cell-like leukemia led to a decrease in cell growth, while overexpression of PIWIL2 in mouse bone marrow cells increased cell proliferation [91]. PIWIL2 overexpression in spontaneously immortalized human keratinocytes was associated with increased expression of pluripotency factors such as c-MYC, pSTAT3, cyclin D1, Bcl-2, β-catenin, N-cadherin, vimentin, Slug, and Snail, reduced expression of p53 and p21 and E-cadherin, and phenotypic changes consistent with EMT [94]. TGF β1 induced EMT in CSCs, which was associated with an increase in PIWIL2 expression and a decrease in Latexin, a negative stem cell regulatory gene [78].

Breast cancer cells overexpressing PIWIL2 formed embryonic stem-like colonies in vitro and displayed a lower apoptosis rate compared with PIWIL2-negative cells. In line with that, PIWIL2 downregulation increased apoptosis accompanied by a decrease in the expression of antiapoptotic and proliferation markers such as STAT3/Bcl-XL/cyclin D1 [12]. PIWIL2 downregulation in cervical cancer cells resulted in reduced proliferation and invasion. The volume of tumors from PIWIL2 knockdown cells was smaller than in the control group. Opposite effects were seen with PIWIL2 overexpression [94].

The mechanism(s), how PIWIL2 contributes to tumor progression and maintenance of a stem cell-like phenotype are largely unknown, but a study found that the CpG island in the promoter of Latexin, a negative stem cell regulatory gene, was more frequently methylated in PIWIL2^+^ breast CSCs [78].

## piRNAs and PIWI proteins as possible biomarkers in cancer

Poorly differentiated and aggressive epithelial cancers often display a deregulation of gene sets that are characteristic of stem cells [95]. In line with that, several studies have reported that cancer stem cell markers are potential diagnostic and prognostic biomarkers [76]. Dysregulated expression of piRNAs and PIWI proteins has been observed in various cancer types. Even though much remains unclear about their role, several studies have reported their association with poor prognosis and assessed their potential use as biomarkers in cancer (Tables 2 and 4). In addition to tissue levels, piRNAs in biological fluids such as serum and urine have also been proposed as potential non-invasive biomarkers (Table 3).

**Table 2:** Expression of piRNAs in cancer tissue compared to non-tumorous tissue and their association with clinicopathological data

**piRNA**	**Cancer type**	**piRNA expression**	**Detection method**	**Association of higher piRNA expression with**		**Notes, references**
				**Survival**	**Metastasis**	
**piR-Hep1**	Hepatocellular carcinoma	Increased	NGS, RT-qPCR			[96]
**piR-001773**	Prostate cancer	Increased	NGS, RT-qPCR		↑	[97]
**piR-017184**	Prostate cancer	Increased	NGS, RT-qPCR		↑	[97]
**piR-017724**	Hepatocellular carcinoma	Decreased	RT-qPCR	↑		Lower expression associated with stage III-IV tumors and poor survival [98].
**piR-021285**	Breast cancer	Not evaluated				Single nucleotide polymorphism (SNP)- rs1326306G > T in piR-021285 is associated with increased risk of breast cancer [99].
**piR-18**	Colorectal cancer	Decreased	RT-qPCR			[100]
**piR-651**	Breast cancer	Increased	RT-qPCR			[101]
	Lung cancer (NSCLC)	Increased	RT-qPCR, Northern Blot, in situ hybridization			[102]
	Hodgkin lymphoma		RT-qPCR			Higher expression in affected lymph nodes associated with limited response to first line therapy, shorter disease-free survival and overall survival (OS) [103].
**piR-823**	Multiple myeloma	Increased	RT-qPCR			Higher expression correlates with advanced stages [55].
	Colorectal cancer	Increased	RT-qPCR			Higher expression correlates with advanced stages [104, 105].
	Gastric cancer	Decreased	RT-qPCR			[106]
	Esophageal cancer	Increased	RT-qPCR		↑	Higher expression associated with lymph node metastasis [107].
	Renal cell carcinoma	Decreased	RT-qPCR	↓		Lower expression compared to healthy tissue. However, higher expression correlates with shorter disease-free survival [108].
**piR-1245**	Colorectal cancer	Increased	NGS	↓	↑	Expression correlates with metastasis, stage of the disease and lower OS [109].
**piR-1742**	Renal cell carcinoma	Increased	Microarray	↓	↑	Increased expression is associated with high tumor grade, advanced stage of the disease and lower survival. Downregulation led to lower proliferation, migration, enhanced apoptosis and tumor growth in vivo. piR-1742 affects cancer cell behavior via the USP8/MUC12 axis [110].
**piR-2158**	Breast cancer	Decreased	NGS, RT-qPCR			PiR-2158 is decreased in ALDH^+^ cancer stem cells (CSC) compared to ALDH- cancer cells. Upregulation led to lower cell proliferation, migration and suppression of EMT and stemness markers [81].
**piR-4987**	Breast cancer	Increased	NGS, RT-qPCR		↑	Higher expression associated with lymph node metastasis [111].
**piR-8041**	Glioblastoma	Decreased	Microarray, RT-qPCR			[112]
**piR-9491**	Glioblastoma	Decreased	NGS, RT-qPCR			[113]
**piR-12488**	Glioblastoma	Decreased	NGS, RT-qPCR			[113]
**piR-13643**	Papillary thyroid carcinoma	Increased	NGS, RT-qPCR			[114]
**piR-14633**	Cervical cancer	Increased	NGS, RT-qPCR			piR-14633 involved in the regulation of METTL14/CYP1B1 [115].
**piR-18849**	Colorectal cancer	Increased	NGS, RT-qPCR		↑	Associated with low degree of differentiation and lymph node metastases [116].
**piR-19521**	Colorectal cancer	Increased	NGS, RT-qPCR		none	Associated with low degree of differentiation [116].
**piR-20365**	Breast cancer	Increased	NGS, RT-qPCR			[111]
**piR-20485**	Breast cancer	Increased	NGS, RT-qPCR			[111]
**piR-20582**	Breast cancer	Increased	NGS, RT-qPCR			[111]
**piR-21238**	Papillary thyroid carcinoma	Increased	NGS, RT-qPCR			[114]
**piR-25783**	Ovarian cancer	Increased	ISH	↓	↑	High expression in cancer tissue associated with high-grade serous ovarian cancer, advanced stage, omentum and appendix metastasis and worse overall survival. Ovarian cancer cells secrete piR-25783 in exosomes contributes to the formation of a premetastatic niche by activating the TGF-β/SMAD2/SMAD3 pathway in omentum fibroblasts [117].
**piR-30188**	Gliomas	Decreased	Microarray, RT-qPCR			[118]
**piR-30473**	DLBCL	Increased	Microarray	↓		Higher expression associated with low OS [119].
**piR-30924**	Clear cell renal cell carcinoma	Increased	Microarray			Upregulation is associated with lymph node metastasis. HIgher expression in primary tumor correlates with recurrence and shorter OS [120].
**piR-31115**	Clear cell renal cell carcinoma	Increased	NGS, RT-qPCR			[121]
**piR-31470**	Prostate cancer	Increased	RT-qPCR			piR-31470 and PIWIL4 contribute to the inactivation of GSTP1 expression via epigenetic modifications [122].
**piR-34536**	Clear cell renal cell carcinoma	Decreased	RT-qPCR	↑		Derived from mitochondrial DNA, lower levels associated with worse cancer specific and OS independent of age, grade and pT and M stage [123].
**piR-34871**	Lung cancer	Variable expression	RT-qPCR			[124]
**piR-35127**	Lung cancer	Variable expression	RT-qPCR			[124]
**piR-36249**	Testicular cancer	Decreased	RT-qPCR			[125]
**piR-36712**	Breast cancer	Decreased	RT-qPCR	↑	↓	Low levels associated with worse PFS and lymph node metastasis, piR-36712 functions as a tumor suppressor. It targets SEPW1P, a retroprocessed pseudogene of SEPW1 and thus inhibits SEPW1 through competition with its regulators miR-7 and miR-324 leading to increased p53, p21 and E-cadherin expression and downregulation of Slug [126].
**piR-38756**	Clear cell renal cell carcinoma	Increased	Microarray	↓	↑	Upregulation is associated with lymph node metastasis. Higher expression in primary tumor correlates with recurrence and shorter OS [120].
**piR-46545**	Lung cancer	Variable expression	RT-qPCR			[124]
**piR-51810**	Clear cell renal cell carcinoma	Decreased	RT-qPCR	↑		Derived from mitochondrial DNA, lower levels associated with worse PFS, cancer specific and OS independent of age, grade and pT and M stage [123].
**piR-52200**	Lung cancer	Variable expression	RT-qPCR			[124]
**piR-54265**	Colorectal cancer	Increased	RT-qPCR	↓		Tissue levels of piRNA-54265 are higher in advanced and metastatic colorectal cancer and are associated with shorter PFS and OS. piRNA-54265 binds PIWIL2 and via activation of STAT3 signaling promotes the proliferation, metastasis and chemoresistance of colorectal cancer cells [73].
**piR-55490**	Lung cancer	Decreased	RT-qPCR	↓		piR-55490 targets 3'UTR of the mTOR mRNA [65].
**piR-57125**	Clear cell renal cell carcinoma	Decreased	NGS, RT-qPCR, Microarray	↑	↓	Lower expression associated with lymph node metastasis. Lower expression correlates with recurrence and shorter OS [120, 127].
**piR-164586**	Lung cancer (NSCLC)	Increased	NGS, RT-qPCR			Low expression associated with stage II and III [128].
**piR-211106**	Lung adenocarcinoma	Decreased	NGS, RT-qPCR			[129]

*NGS* Next Generation Sequencing, *RT-qPCR* Reverse Transcription quantitative PCR, *DLBCL* Diffuse large B cell lymphoma, *NSCLC* Non-Small Cell Lung Cancer

**Table 3:** piRNA levels in serum/plasma and urine of cancer patients compared to healthy controls and their association with clinicopathological data

**piRNA**	**Cancer type**	**piRNA level**	**Detection method**	**Association of higher piRNA level with**		**Notes, references**
				**Survival**	**Metastasis**	
**piR-001311**	Colorectal cancer	Decreased in serum	NGS, RT-qPCR			[130]
**piR-002468**	Prostate cancer	Increased in extracellular vesicles in urine	NGS, RT-qPCR			[131]
**piR-004153**	Colorectal cancer	Decreased in serum	NGS, RT-qPCR			[130]
**piR-004800**	Multiple myeloma	Increased in exosomes	NGS, RT-qPCR			piR_004800 involved in the regulation of PI3K/Akt/mTOR pathway [132].
**piR-004918**	Gastric cancer	Increased in exosomes in serum	NGS, RT-qPCR		↑	Associated with highly metastatic tumors [133].
**piR-017723**	Colorectal cancer	Decreased in serum	NGS, RT-qPCR			[130]
**piR-017724**	Colorectal cancer	Decreased in serum	NGS, RT-qPCR	↑		Lower level associated with shorter PFS and OS [130].
**piR-018569**	Gastric cancer	Increased in exosomes in serum	NGS, RT-qPCR			[133]
**piR-019308**	Gastric cancer	Increased in exosomes in serum	NGS, RT-qPCR		↑	Associated with highly metastatic tumors [133].
**piR-020450**	Colorectal cancer	Increased in serum	NGS, RT-qPCR	↓		[134]
**piR-020619**	Colorectal cancer	Increased in serum	NGS, RT-qPCR	↓		[134]
**piR-020365**	Colorectal cancer	Decreased in serum	NGS, RT-qPCR			[130]
**piR-651**	Hodgkin lymphoma	Decreased in serum	RT-qPCR			A trend for upregulation of serum levels of piR-651 in a small cohort of patients with complete remission after therapy [103].
**piR-823**	Colorectal cancer	Increased in serum	RT-qPCR			Higher expression associated with advanced stages [105].
	Renal cell carcinoma	Increased in serum and urine	RT-qPCR			[108]
**piR-1089**	Neuroblastoma	Increased in exosomes in serum	NGS, RT-qPCR		↑	Inhibition of piR-1089 led to lower cell proliferation and migration [135].
**piR-158533**	Prostate cancer	Increased in extracellular vesicles in urine	NGS, RT-qPCR			[131]
**piR-162725**	Pancreatic cancer	Increased in serum	NGS, RT-qPCR			[136]
**piR-28876**	Colon cancer	Decreased in serum	NGS, RT-qPCR			[137]
**piR-349843**	Prostate cancer	Increased in extracellular vesicles in urine	NGS, RT-qPCR			[131]
**piR-382289**	Prostate cancer	Increased in extracellular vesicles in urine	NGS, RT-qPCR			[131]
**piR-54265**	Colorectal cancer	Increased in serum	RT-qPCR	↓		Plasma piR-54265 levels increase with tumor stage and are associated with worse survival and resistance to neoadjuvant chemotherapy [73]. piR-54265 is specific for colorectal cancer, levels in serum decrease after tumor resection and increase after tumor relapse, suggesting its utility for clinical surveillance of cancer patients [138]. Increased levels of piR-54265 are associated with future colorectal cancer diagnosis [138].
**piR-5937**	Colon cancer	Decreased in serum	NGS, RT-qPCR			[137]

*NGS* Next Generation Sequencing, *RT-qPCR* Reverse Transcription quantitative PCR

### piRNAs in cancer

#### piR-823

In multiple myeloma (MM), piR-823 was described as upregulated and related to the clinical stage of the disease. Silencing of piR-823 in MM cells leads to the dysregulation of the cell cycle, apoptosis, and reduced tumorigenicity in vivo. Expression of the DNA methyltransferases DNMT3A and DNMT3B positively correlates with piR-823. Moreover, downregulation of piR-823 lowers the expression of DNMT3A and DNMT3B, resulting in the activation of tumor suppressor genes such as p16, which were silenced by methylation. Regarding its biomarker potential in MM, piR-823 is strongly correlated with the advanced stages of the disease [55]. The expression of piR-823 is particularly pronounced in myeloid-derived suppressor cells (MDSCs), and its inhibition results in a reduction of the myeloma stem cell phenotype, which is sustained by the MDSCs [139].

In squamous esophageal carcinoma, higher expression of piR-823 was associated with lymph node metastases and higher expression of DNMT3B, supporting their functional relationship seen in MM [107]. In colorectal cancer (CRC), most studies described piR-823 as significantly upregulated. Its inhibition in CRC cells results in reduced proliferation, cell cycle arrest in the G1 phase, and the induction of apoptosis. On the other hand, overexpression of piR-823 in normal epithelial cells promotes their proliferation. piR-823 enhanced the transcriptional activity of HSF1, a shared transcription factor for heat shock proteins, leading to increased expression of HSP27, HSP60, and HSP70 [104]. Inhibition of piR-823 in CRC also promotes mitophagy. piR-823 promotes the ubiquitination and subsequent proteasomal degradation of PINK1, thereby suppressing PINK1-Parkin-mediated mitophagy. Its inhibition led to mitochondrial dysfunction and loss [140]. The use of piR-823 as a biomarker is supported by the findings of its elevated levels in serum in CRC patients and its correlation with the stage of the disease [105].

A different trend in the expression of piR-823 is observed in gastric cancer and renal cell carcinoma (RCC). piR-823 is significantly downregulated in gastric cancer compared to non-tumorous tissue and has suppressive effects on cancer cells in vitro and in vivo [106]***.*** piR-823 is also downregulated in RCC; however, its levels are higher in the serum and urine of RCC patients. Interestingly, higher expression of piR-823 is associated with shorter disease-free survival and a more advanced stage in RCC [108].

In summary, piR-823 acts as a tumor suppressor or promoter depending on the tumor type. In most studies its upregulation has been observed, with the exception of gastric cancer and RCC.

#### piR-651

In breast carcinoma, piR-651 is highly expressed. Overexpression of piR-651 promotes cell proliferation and blocks apoptosis. Higher expression of piR-651 is accompanied by higher expression of oncogenes such as MDM2, CDK4 and Cyclin D1. Functionally, piR-651 in a piRISC with PIWIL2 promotes PTEN promoter methylation by DNMT1 [101]. Increased expression of piR-651 was reported in non-small cell lung carcinoma, where it is associated with tumor progression. Upregulation of piR-651 promotes cancer cell growth and capacity to metastasize [102, 141]. Expression of piR-651 is higher in the tumor tissue in Hodgkin lymphoma and is associated with poor response to first line chemotherapy, shorter disease-free survival as well as shorter overall survival [103].

#### piR-39980

The pathogenetic role of piR-39980 seems to be dependent on the cancer type. In neuroblastoma, piR-39980 expression correlated with the resistance to doxorubicin, its upregulation promoted tumor progression and its inhibition caused induction of senescence [142]. Similarly, in osteosarcoma cells, piR-39980 was upregulated and promoted proliferation, migration and invasiveness, while its inhibition caused induction of apoptosis, chromatin condensation, γ-H2AX-accumulation and decreased invasion and migration. SERPINB1 was identified as a potential molecular target of piR-33980 [143]. In contrast to these tumor promoting effects, piR-39980 attenuated proliferation, invasiveness, colony formation, migration and induced apoptosis by targeting RRM2 in fibrosarcoma. piR-39980 was less expressed in doxorubicin-resistant fibrosarcoma cells and its inhibition led to doxorubicin resistance [144, 145].

#### piR-57125

Two groups described piR-57125 as significantly downregulated in RCC. In addition, lower expression of piR-57125 was associated with metastatic RCC, tumor recurrence and decreased overall survival. piR-57125 upregulation suppressed metastasizing in vivo and the tumor suppressive effect was mediated by the downregulation of the CCL3 chemokine, which led to decreased activation of the AKT/ERK pathway [120, 127].

### PIWI proteins in cancer

#### PIWIL1

Under physiological conditions, PIWIL1 is expressed in spermatocytes and spermatids [11] and CD34^+^ hematopoietic progenitor cells in humans [146]. Ectopic expression of PIWIL1 has been described in many tumor types such as colorectal carcinoma, pancreatic cancer, gliomas, gastric cancer and endometrial cancer (Table 4).

**Table 4:** Expression of PIWI proteins in cancer compared to non-tumorous tissue and their association with clinicopathological data

**PIWI**	**Cancer type**	**PIWI expression**	**Detection method**	**Association of higher PIWI expression with**		**Notes, references**
				**Survival**	**Metastasis**	
**PIWIL1**	Colorectal cancer	Increased [147–150]	RT-qPCR [148, 150], RT-PCR [149], WB [150], IHC [147, 149, 150]	↓ [147, 149]	↑ [148, 149] / none [147]	PIWIL1 is an independent prognostic factor in patients without lymph node metastasis, but not in patients with lymph node metastasis [147]. More than two-fold higher expression of *PIWIL1* mRNA in 16/46 (34.8%) of tumor tissues compared to healthy surrounding tissue [148]. PIWIL1 detected in 72/225 (32%) of tumors [147].
	Pancreatic cancer	Increased [72]	IHC [72]	↓ [72]	↑ [72]	
	Gliomas	Increased [74, 84, 151]	RT-qPCR [74], RT-PCR [151], WB [151], IHC [84, 151]	↓ [151]		PIWIL1 expression associated with higher tumor grade [151].
	Gastric cancer	Increased [74, 152, 153]	RT-qPCR [74], RT-PCR [152], IHC [74, 152, 153]	↓ [74, 153]	↑ [74] / none [153]	*PIWIL1* mRNA expression associated with poor survival in patients with poorly differentiated or mixed-classification tumors and patients who underwent 5-fluorouracil-based adjuvant therapy [74].
	Endometrial cancer	Increased[86]	RT-qPCR [86], IHC[86]		↑[86]	
	Seminoma	Increased [11] / Decreased [154]	RT-qPCR [154], RT-PCR [11]			No significant change in *PIWIL1* mRNA in other germ cell testicular tumors and stromal cell testicular tumors [11, 154].
	Breast cancer	Increased [155, 156] / variable expression [157]	RT-qPCR [155–157], WB [155, 156]	↓ [156]	↑ [155]	*PIWIL1* mRNA expression associated with higher tumor grade [155].
	Lung cancer (NSCLC)	Increased [13, 82, 158] / no change [159]	RT-qPCR [82, 158], RT-PCR [13, 159], WB [158, 159], IHC [159]	↓ [82]		*PIWIL1* mRNA expressed in 11/71 (15.5%) of the tumor tissues and not detectable in the healthy adjacent tissue. *PIWIL1* is an independent prognostic factor [82].
	Hepatocellular carcinoma	Increased [160–162] / Not detected [96]	RT-qPCR [96, 161, 162], RT-PCR [160], WB [160, 161], IHC [160, 162]	↓ [160, 162]	↑ [160, 162]	
	Renal cell carcinoma	Increased [163] / Decreased [164]	RT-qPCR [164], IHC [163]	↑ [164]/↓ [163]	↓ [163]	PIWIL1 detected in 75/265 (28.3%) and 51/345 (14.8%) of tumors in two independent cohorts, no expression in adjacent tissues. PIWIL1 expression associated with higher tumor grade and worse survival [163]. *PIWIL1* mRNA expression associated with lower tumor grade and higher piR-823 expression [164].
**PIWIL2**	Colorectal cancer	Increased [165, 166]	WB [165], IHC [165, 166]	↓ [165, 166]	↑ [165, 166] / none [166]	PIWIL2 expression associated with low degree of differentiation and perineural, but not lymphatic or venous invasion. No association with lymph node involvement and distant metastasis. PIWIL2 was not an independent prognostic factor [166].
	Gliomas	Increased [167]	WB [167], IHC [167]	↓ [167]		PIWIL2 expression associated with higher tumor grade [167].
	Lung cancer (NSCLC)	Increased [159] / Decreased [82]	RT-qPCR [82, 159], WB [159], IHC [159]	↓ [159]		
	Gastric cancer	Increased [153] / No change [74]	RT-qPCR [74], IHC [153]	↓ [153]	none [153]	PIWIL2 is not an independent prognostic factor [153].
	Prostate cancer	Increased [168]	RT-qPCR [168], IHC [168]		↑ [168]	
	Breast cancer	Increased [12, 78, 156, 157, 169]	RT-qPCR [156, 157], WB [78, 156], IHC [12, 78, 169]	none [12, 156]	↑ [78]	*PIWIL2* mRNA expression was significantly higher in 2/20 (10%) of the tissues in one of the studies [157]. PIWIL2 was higher in 334/1086 (30.7%) of the tissues in another study [78].
	Soft tissue sarcoma	Increased [170]	RT-qPCR [170]	↑ [170]		High *PIWIL2* mRNA expression associated with high *PIWIL4* mRNA expression. No association with *PIWIL3* [170].
	Cervical cancer	Increased [94, 171]	WB [94], IHC [94, 171]			
	Hepatocellular carcinoma	Increased [96]	RT-qPCR [96]			
	Seminoma	Increased [172] / Decreased [154]	RT-qPCR [154], RNA microarray [172]			No change or decrease in *PIWIL2* mRNA expression in non-seminoma testicular tumors [154, 172].
	Renal cell carcinoma	No change [164]	RT-qPCR [164]	↑ [164]		*PIWIL2* mRNA associated with lower tumor grade [164].
	Non-melanoma skin cancer	No change [173]	IHC [173]			Concordant results in basal cell and squamous cell carcinomas [173].
	Bladder cancer	Not detected [14]	RT-qPCR [14]			
**PIWIL3**	Gastric cancer	Increased [153, 174] / No change [74]	RT-qPCR [74, 174], IHC [153]	none [153]	↑ [174] / none [153, 174]	*PIWIL3* mRNA expression associated with presence of lymph node metastasis in diffuse-type but not in intestinal-type gastric cancer [174].
	Soft tissue sarcoma	Increased [170]	RT-qPCR [170]	none [170]		
	Renal cell carcinoma	No change [164]	RT-qPCR [164]	none [164]		
	Lung cancer (NSCLC)	Increased [82] / No change [159]	RT-qPCR [82, 159], WB [159], IHC [159]			*PIWIL3* mRNA detected in 5/71 (7%) of the tumor tissues, no expression in healthy adjacent tissue [82].
	Breast cancer	No change [156]	RT-qPCR [156], WB [156]			
	Hepatocellular carcinoma	No change [96]	RT-qPCR [96]			
	Gliomas	Decreased [118]	WB [118]			PIWIL3 expression associated with lower tumor grade [118].
	Seminoma	Decreased [154]	RT-qPCR [154]			
**PIWIL4**	Soft tissue sarcoma	Increased [170]	RT-qPCR [170]	↑ [170]		High *PIWIL4* mRNA expression associated with high *PIWIL2* mRNA expression. No association with *PIWIL3* [170].
	Gastric cancer	Increased [153] / No change [74]	RT-qPCR [74], IHC [153]	none [153]	↑ [153]	
	Cervical cancer	Increased [175]	RT-qPCR [175]			
	Hepatocellular carcinoma	Increased [96]	RT-qPCR [96]			*PIWIL4* mRNA expression not associated with piR-Hep1 expression [96].
	Breast cancer	Increased [157] / no change [156]	RT-qPCR [156, 157], WB [156]			
	Renal cell carcinoma	No change [164]	RT-qPCR [164]	↑ [164]		*PIWIL4* mRNA expression associated with lower tumor grade [164].
	Lung cancer (NSCLC)	No change [159] / Decreased [82]	RT-qPCR [82, 159], WB [159], IHC [159]	↑ [82]		
	Seminoma	Decreased [154]	RT-qPCR [154]			Decrease in *PIWIL4* mRNA expression in non-seminoma testicular tumors [154].

*RT-qPCR* Reverse Transcription quantitative PCR, *WB* Western Blot, *IHC* Immunohistochemistry, *NSCLC* Non-Small Cell Lung Cancer

In colorectal carcinoma, four independent studies reported increased levels of PIWIL1 at transcript and protein level in cancer tissue compared to non-tumorous tissue [147–150]. Liu et al*.* reported no correlation between PIWIL1 expression and TMN stage, depth of invasion or lymph node metastasis and showed no significant difference in disease-free survival (DFS) between PIWIL1-positive and PIWIL1-negative colorectal tumors. However, when patients were stratified, PIWIL1 was an independent negative prognostic indicator in patients without lymph node metastases [147]. In contrast, Sun et al*.* described an association between high PIWIL1 expression and more advanced TNM stage, lymphovascular invasion, lymph node metastasis, and poor tumor differentiation in a cohort of 110 colorectal carcinoma patients. Moreover, expression of *PIWIL1* mRNA was shown to be an independent progostic factor for DFS and overall survival (OS) [149]. Accordingly, Raeisossadati *et. al.* showed an association of *PIWIL1* mRNA expression with tumor invasion depth and tumor stage [148]. Studies in gliomas, endometrial, pancreatic and gastric cancer have also shown an association of higher levels of PIWIL1 with more aggressive disease [72, 74, 86, 151, 153].

Although some works in breast cancer, hepatocellular carcinoma and non-small cell lung cancer showed that upregulation in PIWIL1 expression is associated with metastasis and/or poor survival, discrepant data have been published. Two studies showed significantly higher levels of PIWIL1 in breast cancer compared to non-tumorous tissue and reported a positive association with tumor size, lymph node metastasis and histological grade and negative association with tumor-specific survival rate [155, 156]. Nevertheless, increased levels of PIWIL1 were not observed in the majority of 20 breast cancer tissues analyzed in an independent study [157]. Similarly, three studies in hepatocellular carcinoma described increased levels of PIWIL1 in tumor specimens compared to peritumoral tissues [160–162]. Patients with high PIWIL1 had larger tumors, higher incidence of intrahepatic and lymph node metastasis and lower DFS and OS showing that PIWIL1 is an independent prognostic factor in hepatocellular carcinoma [160, 162]. Surprisingly, Law et. al. did not detect *PIWIL1* mRNA in hepatocellular carcinoma tissues [96]. In non-small cell lung carcinoma, three independent studies described an increased expression of PIWIL1 in tumor tissue [13, 82, 158], whereas no significant difference in PIWIL1 expression was reported by another group [159]. Similar contradictory data were reported in renal cell carcinoma. A study in two large patient cohorts detected PIWIL1 in 15% and 28% of the patients and demonstrated its association with higher Fuhrman grade, more advanced tumor stage, presence of distant metastasis and shorter cancer-specific survival [163]. Nevertheless, Iliev et. al. demonstrated a decreased expression of *PIWIL1* mRNA in renal cancer which was associated with a more advanced clinical stage, higher Fuhrman grade and worse OS [164].

Given the current evidence, PIWIL1 could be a potential prognostic biomarker in some cancer types, such as colorectal carcinoma. In several other cancer types, validation studies are still limited, or their results are contradictory.

#### PIWIL2

Similar to PIWIL1, PIWIL2 seems to be predominantly expressed in spermatogonia and spermatocytes under physiological conditions in humans; in addition one study described its expression in keratinocytes and skin adnexa [15, 172, 173]. An increasing number of studies have reported aberrant expression of PIWIL2 in cancer. Most of the analyzed cancer types, including colorectal carcinoma, gliomas, gastric cancer, prostate cancer, breast cancer, soft tissue sarcoma, cervical cancer and hepatocellular carcinoma, showed an upregulation of PIWIL2 (Table 4).

In colorectal cancer, two independent groups observed the presence of PIWIL2 in most of the analyzed tumor tissues in comparison to weak or no positivity in healthy tissues. Li *et. al.* proposed PIWIL2 as a novel prognostic marker to predict distant metastases in colon cancer patients who underwent radical colectomy given the association between PIWIL2 expression and the presence of distant metastasis, lymph node involvement, and clinical stage. Oh *et. al.* did not report differences in lymph node involvement, distant metastasis and lymphatic or venous invasion, but described a lower degree of differentiation and higher degree of perineural invasion in patients with high PIWIL2 expression. Both studies showed a better patient outcome when PIWIL2 was weakly expressed or absent [165, 166]. Similarly, low expression of PIWIL2 has been linked to better patient survival in other cancers such as glioma and gastric cancer [153, 167]. The data in non-small cell lung cancer are conflicting with one study reporting higher expression of *PIWIL2* mRNA in the tumor and its association with poor survival [159], while another study showed a downregulation of *PIWIL2* mRNA [82].

In breast cancer, several studies showed an upregulation of PIWIL2 in tumor tissue with highest levels in invasive carcinomas as compared to benign or in situ carcinomas, but no significant differences in patient outcome between PIWIL2 low and high-expressing groups [12, 156, 157, 169]. Half of the breast cancer patients with PIWIL2 expression developed distant metastases within 5 years in contrast with only 13% of the patients without PIWIL2 expression [78].

The only cancer type exhibiting better cancer-specific survival with higher levels of *PIWIL2* mRNA is soft tissue sarcoma. Interestingly, in this tumor type PIWIL2 expression negatively correlated with the stem cell markers POU5F1 and NANOG [170].

In summary, PIWIL2 is expressed in several cancer types, however, data on its possible use as a biomarker are currently limited.

#### PIWIL3

Compared to other members of the PIWI subfamily, limited data have been published regarding PIWIL3 in humans. PIWIL3 seems to be expressed in the ovary and possibly testis, although not all studies were able to confirm this [15, 176–180]. No expression was reported in somatic tissues [15].

Only one study in soft tissue sarcoma reported higher levels of *PIWIL3* mRNA in the tumors [170]. Studies in renal cell carcinoma, breast cancer, hepatocellular carcinoma, gliomas and seminomas showed no change or a decrease in expression compared to non-tumorous tissue [96, 118, 154, 156, 164] (Table 4). Conflicting data have been published in gastric and non-small cell lung cancer [74, 82, 153, 159, 174]. None of the studies showed an association with the presence of distant metastasis and patient survival [153, 164, 170, 174]. Given the limited data currently available, PIWIL3 does not seem to be a candidate biomarker in cancer.

#### PIWIL4

PIWIL4 is the only PIWI protein that is ubiquitously expressed in somatic tissues and enriched in testicular tissue, specifically in spermatogonia [15, 176, 181, 182]. A limited number of studies analyzed PIWIL4 expression in cancer. PIWIL4 is increased in soft tissue sarcoma, cervical cancer and hepatocellular carcinoma compared to non-tumorous tissue. On the other hand, renal cell, non-small cell lung cancer and seminoma exhibit no change or a decrease in PIWIL4 expression (Table 4). Conflicting data on expression of PIWIL4 are available in breast cancer and gastric [74, 153, 156, 157]. Surprisingly, most of the studies analyzing effect on survival reported higher levels of PIWIL4 as a favorable prognostic factor [82, 164, 170]. The scarce information about PIWIL4 in human cancer does not provide support for its use as a biomarker in cancer.

## Limitations of current studies evaluating the role of the piRNA-PIWI axis in cancer

piRNA and PIWI proteins have been proposed to be promising biomarkers in various types of cancer, in some cases demonstrating a robust ability to predict survival and/or metastatic spread. However, there are several uncertainties and technological challenges that make their diagnostic and prognostic use problematic.

Among the four PIWI proteins expressed in humans, PIWIL1 and PIWIL2 seem to be the most promising candidates to be prognostic biomarkers in cancer [149, 165]. Their contribution to tumor progression may be explained by the maintenance of the CSC subpopulation since PIWIL1 and PIWIL2 have been associated with expression of CSC markers and increased tumor formation, migration and invasion [84, 86, 94] (Fig. 2). Although the increased expression of PIWI proteins is frequently not accompanied by deregulation of piRNAs, their piRNA-independent functions may contribute to cancer progression [71, 72, 74].

However, the connection between elevated expression of PIWI proteins and poor patient outcome in a particular cancer type has not been robustly validated by independent investigations. Some works reported discrepant results for the expression of PIWI proteins in malignant tissues compared to non-tumorous tissue [96, 162]. This may be due to rather small patient cohorts and difficulties in detecting PIWI proteins. Currently available antibodies have not been extensively validated and may lack the sensitivity for reliable detection of low levels of PIWI proteins. According to The Human Protein Atlas, a commercially available antibody reliably stained testicular tissue, whereas it did not detect PIWIL1 in most of the cancer tissues. Strikingly, PIWIL1 was not detectable in 10 out of 11 colorectal cancer tissues, despite three independent studies reporting positive staining in large proportion of the tumors [147, 149, 150]. Moreover, none of the reviewed studies validated the immunohistochemistry findings with an additional antibody, which is a recommended practice to ensure specific detection of the target [183].

The studies proposing piRNAs as promising biomarkers in cancer also come with notable limitations. Most of the published works performed an explorative phase to select one or a few aberrantly expressed piRNAs to be further examined as candidate biomarkers. Surprisingly, some aberrantly expressed piRNAs have been documented only by a single research group and not reported by others. High degree of inconsistency between individual studies may be caused by inadequate size of patient cohorts [97, 112, 124], exploratory phases being only performed on cell lines [65, 123, 184] and the absence of validation [112, 127]. In addition, reasons why particular piRNAs have been selected are not clearly described in some studies [65, 107].

Very limited information is available on the biogenesis of piRNAs in cancer tissues. A fundamental question is whether piRNAs are in fact so widely expressed in cancer tissues as is currently assumed. A study by Tosar et al. revealed that most of the piRNAs reported to be expressed in somatic tissues (mouse brain and human cancer) seem to be fragments of other molecules such as tRNAs, snoRNAs, pre-miRNAs, YRNAs, rRNAs or snRNAs. These fragments lack the characteristic features of piRNAs, such as a 1U bias of primary piRNAs and 10A bias of secondary piRNAs. Generation through the piRNA biogenesis pathway leading to the incorporation of the molecules into piRISCs is a defining feature of piRNAs [3]. However, many of the reported molecules arise from precursors that are unlikely to undergo piRNA biogenesis [185] and there is little evidence whether they in fact interact with PIWI proteins. Immunoprecipitation experiments that could confirm these interactions are scarcely reported, possibly due to the lack of highly specific antibodies against PIWI proteins. Nevertheless, the uncertain nature of the reported RNA molecules does not automatically disqualify them from being good biomarkers in cancer. Stability of expression, robust differences in expression between examined conditions and practical usability are the key properties of quality biomarkers, which could be true for many molecules listed in Tables 2 and 3.

Some limitations of the current studies relate to the still very unclear biogenesis of piRNAs and biological functions of the piRNA-PIWI axis in cancer. In germline cells, piRNA clusters are expressed and processed into individual piRNAs that target the same TE sequence from which they originated. This cluster-based silencing is the main and robust tool for efficient tackling of TE elements in the germline [31, 35, 56]. Given their coordinated biogenesis and mechanism of action, it is still uncertain whether dysregulation of a single piRNA molecule in cancer is sufficient to have functional consequences and what would be the mechanism leading to this selective amplification of a particular piRNA from the whole cluster. Most of the studies focus on just one or a few dysregulated piRNAs, not allowing to unravel the cooperative action of multiple piRNAs. Cluster-based piRNA studies rather than analysis of single piRNA molecules could provide a more accurate insight into the role of the piRNA-PIWI axis in cancer. Also, the use of new technologies enabling more precise analysis of expression in specific cell types, such as single-cell RNA sequencing, could help explain discrepancies and uncertainties that currently exist in the field of piRNA-PIWI protein research in cancer. Compilation of piRNA profiles for specific cell types will be invaluable to determine whether specific piRNA molecules are linked to cancer cells and specifically to cancer stem cells. Analysis of piRNA by single-cell sequencing is still in its beginning, nevertheless a recent study in human oocytes and early embryos provides a new look into the biogenesis and function of piRNAs [186].

## Conclusions

Several studies demonstrate that the piRNA-PIWI axis may play a role in maintaining the undifferentiated phenotype of CSCs. This subpopulation of cancer cells, which is resistant to chemotherapy and radiotherapy, is thought to importantly contribute to tumor recurrence following treatment. If the involvement of the piRNA-PIWI axis in the maintenance of the stem cell phenotype is substantiated, the prospect of targeting piRNAs and/or PIWI proteins emerges as a potential strategy to mitigate the resistance of cancer to treatment. In addition, there is evidence that expression of certain piRNAs and PIWI proteins, in particular PIWIL1 and PIWIL2, is associated with more aggressive clinical course. This may be linked to the fact that aggressive cancers often adopt various programs utilized by stem cells, and suggests that piRNAs and PIWI proteins are potential prognostic factors. Nevertheless, literature evidence for various piRNAs and PIWI proteins needs to be interpreted with caution due to the limitations of currently available studies in this actively evolving field. Studies that improve our understanding of the piRNA-PIWI axis in cancer and comprehensive validation studies confirming the diagnostic, prognostic and predictive value of piRNAs and PIWI proteins are warranted.

## Data Availability

No datasets were generated or analysed during the current study.

## Abbreviations

ALDH: Aldehyde dehydrogenase
APC/C: Anaphase promoting complex/cyclosome
CRC: Colorectal cancer
DLBCL: Diffuse large B cell lymphoma
DFS: Disease-free survival
Egg: Eggless
Mael: Maelstrom
MM: Multiple myeloma
MDSCs: Myeloid-derived suppressor cells
NGS: Next generation sequencing
NSCLC: Non-small cell lung cancer
piRISC: PiRNA-induced silencing complex
piRNAs: PIWI-interacting RNAs
PAZ: PIWI/Argonaute/Zwille
PABP: Poly(A)-binding protein
RCC: Renal cell carcinoma
RT-qPCR: Reverse transcription quantitative PCR
sncRNAs: Small noncoding RNAs
TE: Transposable elements
Wde: Windei
